# In Vitro Antioxidant, Antityrosinase, and Cytotoxic Activities of Astaxanthin from Shrimp Waste

**DOI:** 10.3390/antiox8050128

**Published:** 2019-05-13

**Authors:** Sutasinee Chintong, Wipaporn Phatvej, Ubon Rerk-Am, Yaowapha Waiprib, Wanwimol Klaypradit

**Affiliations:** 1 Department of Fishery Products, Faculty of Fisheries, Kasetsart University, Bangkok 10900, Thailand; sutasinee_c@yahoo.com (S.C.); ffisywp@gmail.com (Y.W.); 2 Expert Center of Innovative Herbal Products, Thailand Institute of Scientific and Technological Research, Pathum Thani 12120, Thailand; wipaporn@tistr.or.th (W.P.); ubon@tistr.or.th (U.R.-A.); 3 Center for Advanced Studies for Agriculture and Food, Kasetsart University Institute for Advanced Studies, Kasetsart University, Bangkok 10900, Thailand

**Keywords:** shrimp astaxanthin, antioxidant property, tyrosinase inhibition, cytotoxicity

## Abstract

Astaxanthin is a potent antioxidant compared with vitamins and other antioxidants. However, astaxanthin extract from shrimp processing waste has not yet been used in cosmetic products. This study aimed to explore the natural astaxanthin from shrimp shells for antioxidant and antityrosinase activities as well as potential toxicity. The antioxidant activities were performed with 2,2-diphenyl-1-picrylhydrazyl (DPPH), 2,2’-azino-bis(3-ethylbenzothiazoline-6-sulfonic acid) (ABTS) radical scavenging, β-carotene bleaching, and singlet oxygen quenching assays. The results revealed that astaxanthin extract demonstrated potent antioxidant activities against DPPH and ABTS radicals, and prevented the bleaching of β-carotene and quenching of singlet oxygen (EC_50_ 17.5 ± 3.6, 7.7 ± 0.6, 15.1 ± 1.9 and 9.2 ± 0.5 μg/mL, respectively). Furthermore, the astaxanthin extract could inhibit tyrosinase activity (IC_50_ 12.2 ± 1.5 μg/mL) and had no toxic effects on human dermal fibroblast cells. These results suggested that shrimp astaxanthin would be a promising dietary supplement for skin health applications.

## 1. Introduction

Astaxanthin is a ketocarotenoid synthesized by plants and microorganisms but is distributed mainly in aquatic animals such as crustaceans, salmon, and trout. It possesses antioxidant activity 10 times stronger than zeaxanthin, lutein, and canthaxanthin [1] and has beneficial effects supporting human health including alleviation of oxidative stress, inhibition of low-density lipoprotein (LDL) oxidation, enhancement of immune response, anti-inflammatory and anti-aging properties, with its major mode of action being a scavenger of reactive oxygen species (ROS) [2].

Shrimp waste is one of the important natural sources of carotenoid; astaxanthin and its esters as the major pigments. Several methods for extraction of astaxanthin have been attempted. Organic solvent has been used to recover the pigment from crustacean processing discards [3]. Alcohol was considered to be appropriate for astaxanthin due to its safety, effectiveness, and easier separation. Ethanolic extract of astaxanthin was used to fortify in yogurt as an optional functional food for consumers [4] and was investigated the anti-inflammatory effect on alveolar macrophages [5]. Nevertheless, the extract from shrimp waste has not been yet studied on skin effect. 

Some studies have been done on skin protective effects by astaxanthin from *Haematococcus pluvialis*. These studies have demonstrated that astaxanthin contributed to maintain moisture of skin and decrease skin roughness, an early stage of wrinkle formation, by inhibiting lipid peroxidation [6] and also effectively inhibiting UVB-induced age spots and atopic dermatitis [7]. Moreover, the effects of astaxanthin in ROS scavengers or inhibitors have been implicated in the treatment of skin pigmentation disorders [8]. Skin pigmentation, such as suntan, blemishes, and freckles, is caused by excessive and uneven production of melanin. It is known that pigmentation in the skin can be prevented by reducing tyrosinase activity, either by inhibiting the synthesis of tyrosinase—which is an important enzyme for melanin synthesis—or by using an antagonist of the substrate for tyrosinase. Pigmentation is also prevented by inhibiting auto-oxidation of dopa and suppressing inflammatory reactions [9]. 

Previous reports showed that astaxanthin could reduce tyrosinase activity and prevent melanin synthesis by inhibiting auto-oxidation of dopa and dopaquinone, and as a result, the amount of melanin was decreased by 40% with no change in cell viability of B16 mouse melanoma cells [10]. Also, it was found that astaxanthin attenuates the stem cell factor (SCF), which downregulates tyrosinase activity and reduces melanin production [11]. In vivo study demonstrated multiple biological activities of astaxanthin to preserve skin health and achieve effective skin cancer chemoprevention [12]. In addition, with respect to safety, no adverse events were observed with oral astaxanthin supplementation [13]. 

These studies suggest skin therapeutic potentials of astaxanthin; however, there is currently no report showing the utilization of astaxanthin from shrimp waste, which is a natural and cheap source for astaxanthin recovery [14]. At present, natural astaxanthin derived from *Haematococcus pluvialis* is the major dietary supplements for humans and animals [15].

In the course of screening such natural substances, in vitro studies must be conducted to determine their efficacy and safety. Therefore, the aim of this study was to investigate the concentration of astaxanthin from shrimp shells which exhibits optimal levels of antioxidant and antityrosinase activities as well as to evaluate the possible cytotoxic effects on human dermal fibroblast cells in order to safely utilize as a functional ingredient for skin health.

## 2. Materials and Methods 

### 2.1. Materials

Fresh white shrimp shells (*Litopenaeus vannamei*) were obtained from a frozen shrimp processing plant in Samut-Sakorn Province, Thailand. The materials were packed into plastic food storage bags with the weight approximately 1 kg and frozen at −20 °C until use. Prior to use, samples were thawed by submerging the bag under running tap water until completely thawed. 

### 2.2. Preparation of Astaxanthin from Shrimp Shells

Astaxanthin in shrimp waste was extracted using ethanol. Five hundred grams of shrimp shells was blended with 1000 mL of ethanol using a Waring laboratory blender (Waring Laboratory Science, Winsted, USA). Shrimp shell residues were vacuum-filtered and evaporated under vacuum at 40 °C, 175 MPa using a rotary evaporator (Büchi Labortechnik AG, Flawil, Switzerland). The resulting concentrate was stored at −20 °C until further analysis.

### 2.3. Analysis of Astaxanthin Extract

The content of astaxanthin from shrimp shells was performed using HPLC analysis as previously described [16]. The astaxanthin extract was dissolved in a dichloromethane/methanol (HPLC grade) (25:75) mixture. The sample was passed through a 0.45 μm filter. Twenty microliters of filtered sample was injected into the HPLC system (LC 20A, Shimadzu, Japan) and eluted through a reversed phase C18 column (Beckman Ultrasphere C18, 250 × 4.6 mm, Phenomenex, Torrance, CA, USA). Chromatography was isocratically performed at 25 °C. The mobile phase consisted of methanol, dichloromethane, acetonitrile, and water (85:5:5:5 *v/v/v/v*), at a flow rate of 1 mL/min. The UV detection of elute was performed at 480 nm. Astaxanthin was qualitatively analyzed by comparing the retention times of standards and their quantifications were done by using a calibration curve.

### 2.4. Antioxidant Properties Assay

#### 2.4.1. Determination of DPPH Free Radical-Scavenging Activity

The radical scavenging activity of astaxanthin was evaluated using a modified 2, 2-diphenyl-1-picryl-hydrazyl (DPPH) radical scavenging assay following previous report [17]. One hundred microliters of the DPPH solution in methanol was added to 100 µL of various concentrations of the astaxanthin in methanol (3–50 µg/mL), then shaken vigorously and allowed to stand in the dark at room temperature for 30 min. The absorbance of the sample solution was measured at 517 nm using a microplate reader (Thermo Fisher Scientific, Inc., Waltham, MA, USA). A curve of astaxanthin concentration against %DPPH was generated to estimate the concentration of astaxanthin needed to cause a 50% reduction of the initial DPPH concentration. This value is known as EC_50_ (efficient concentration when 50% oxidation is achieved, also called oxidation index) and was expressed in units of µg/mL. This assay was carried out in triplicate and the mean values were used to calculate the EC_50_. Ascorbic acid and BHT (butylated hydroxytoluene) were used as positive controls. The scavenging effect on the DPPH inhibition as percentage (%) was calculated according to the equation

%DPPH radical scavenging = [A_control_ − (A_sample_ − A_sample blank_)/A_control_] × 100, (1)

where A_control_, A_sample_, and A_sample blank_ are the absorbance of the DPPH solution in methanol, astaxanthin solution with DPPH, and astaxanthin solution without DPPH, respectively.

#### 2.4.2. Determination of ABTS Radical Scavenging Activity

The ABTS radical scavenging assay was measured following the modified method as previously described [18]. The ABTS radical solution was prepared by mixing 7 mM ABTS and 2 mM K_2_S_2_O_8_ in equal quantities and incubating at room temperature for 16 h in the dark. The solution was diluted with water to obtain an absorbance of 0.70 ± 0.03 units at 734 nm. Two hundred microliters of the working ABTS solution was mixed with 20 μL of the different concentrations of the extract (astaxanthin in ethanol 3–50 µg/mL), the mixture was incubated at room temperature for 6 min, and then absorbance was recorded at 734 nm. Ascorbic acid and BHT were used as positive controls. All determinations were carried out in triplicate and EC_50_ value was expressed as the sample concentration which can quench fifty percent of ABTS radicals. The percentage of ABTS radical scavenging was calculated as

%ABTS radical scavenging = [A_control_ − (A_sample_ − A_sample blank_)/A_control_] × 100, (2)

where A_control_, A_sample_, and A_sample blank_ are the absorbance of the ABTS solution, astaxanthin solution with ABTS, and astaxanthin solution without ABTS, respectively.

#### 2.4.3. Determination of β-Carotene Bleaching (BCB) Activity

Protocol for BCB activity was previously described with slight modifications [19]. A solution of β-carotene/linoleic acid was initially prepared by dissolving 10 mg of β-carotene in 10 mL of chloroform. Two mL of this solution was added to 40 mg of linoleic acid and 400 mg of Tween 40. The chloroform was evaporated off using a rotary evaporator. Then, 100 mL of aerated distilled water was added to the mixture with vigorous shaking. Aliquots of 100 µL of β-carotene/linoleic acid emulsion were added to 100 µL of different concentrations of astaxanthin in ethanol (3–50 µg/mL) in a 96-well plate. The zero time absorbance was measured at 470 nm using a microplate reader. The plates were incubated at 50 °C in a water bath and measurement of absorbance was recorded after 60 min; a blank, absent of β-carotene, was prepared for background deduction. The same procedure was repeated with the synthetic antioxidants, ascorbic acid, and BHT as positive controls. The assay was carried out in triplicate and EC_50_ value was calculated. The percentage antioxidant activity (%AOA) was calculated using the formula

%AOA = [(Ac_(0)_ − Ac_(60)_)/(As_(0)_ − As_(60)_)]/(Ac_(0)_ − Ac_(60)_) ×100, (3)

where Ac_(0)_ is the absorbance of the control at *t* = 0 min, Ac_(60)_ is the absorbance of the control at *t* = 60 min, As_(0)_ is the absorbance of the sample at *t* = 0 min, and As_(60)_ is the absorbance of the sample at *t* = 60 min.

#### 2.4.4. Determination of Singlet Oxygen Scavenging (^1^O_2_) Activity

The formation of singlet oxygen was determined by monitoring p-nitrosodimethylaniline (RNO) bleaching according to a modified method as was previously described [20]. The bleaching of RNO was measured after singlet oxygen was generated by reacting H_2_O_2_ and NaOCl. The reaction mixture contained 50 μL of sample at astxanthin in ethanol various concentrations (3–50 µg/mL), 50 μL of 45 mM phosphate buffer (pH 7.1), 50 μL of 50 mM H_2_O_2_, 50 μL of NaOCl, 50 mM, 50 μL of 50 mM histidine, and 50 μL of 10 μM RNO. The mixture was incubated at 30 °C for 40 min, and the decrease in RNO absorbance was measured at 440 nm. Rutin and quercetin were used as positive controls and the values were expressed as EC_50_. The percentage of singlet oxygen scavenging was calculated from the equation

% Singlet oxygen scavenging = [A_control_ − (A_sample_ − A_sample blank_)/A_control_] × 100, (4)

where A_control_, A_sample_, and A_sample blank_ are the absorbance of the mixture solution, astaxanthin with mixture solution and astaxanthin without mixture solution, respectively.

### 2.5. Determination of Tyrosinase Inhibitory Activity

Tyrosinase inhibitory activity was measured according to the modified dopachrome method [21]. In brief, 50 mL of astaxanthin extract in ethanol at various concentrations (3−50 µg/mL) was applied to a 96-well plate followed by the addition of 50 μL of 20 mM phosphate buffer (pH 6.8) and mushroom tyrosinase (500 units/mL). Each well was mixed and incubated at room temperature for 15 min. Then, 50 μL of 0.85 mM L-3,4-dihydroxyphenylalanine (L-DOPA) was added. After incubation at room temperature for 10 min, the mixture was measured for the difference of the absorbance at 492 nm before and after incubation. Kojic acid and arbutin were used as positive controls and the values were expressed as 50% inhibitory concentration (IC_50_). The percentage of tyrosinase inhibition was calculated as

%inhibition = [(A−B) − (C−D)]/(A−B) ×100,(5)

where A is the absorbance of the blank after incubation, B is the absorbance of the blank before incubation, C is the absorbance of the sample after incubation, and D is the absorbance of the sample before incubation.

### 2.6. Cytotoxicity Assay

#### 2.6.1. Cell Culture

Human dermal fibroblasts (WS1) were purchased from American Type Culture Collection (ATCC, Manassas, VA, USA). The cells were cultured in Dulbecco’s modified Eagle’s medium (DMEM) supplemented with 10% fetal bovine serum (FBS) and 1% L-glutamine and 1% penicillin-streptomycin in 5% CO_2_ environment at 37 °C until they reached a confluence of 70−80%.

#### 2.6.2. Cell Viability

Cell viability was determined using an MTT assay as described by previous report [22]. The cells were seeded at 1 × 10^5^ cells/well in a 96-well plate and were treated with 100 µL of astaxanthin in at various concentrations (5–160 µg/mL), and then the plates were incubated at 37 °C with 5% CO_2_ for 24 h. The test compound was dissolved in dimethyl sulfoxide (DMSO), and the final concentration of DMSO in the medium was 1% *v/v*. For the control, no astaxanthin was added (none). Thereafter, 100 µL of MTT solution was added to each well and incubation performed for 2 h. After 2 h, the formazan crystals were dissolved by adding 100 μL DMSO and the plate was further incubated for 5 min at room temperature. The absorbance was measured at 570 nm using a microplate reader. Percentage of cell viability was calculated by comparing absorbance values of samples with those of the control.

#### 2.6.3. Microscopic Observations

The changes in morphology and detachment of human dermal fibroblasts were observed after incubation with astaxanthin using a Nikon inverted microscope (Nikon Eclipse TS100, Nikon, Japan) equipped with an objective lens (Plan 10/0.25DL/Ph1, Nikon, Japan) of ×10 magnification.

### 2.7. Statistical Analysis

All results are expressed as mean ± SD (standard deviation). Analysis of variance was performed, and significant differences between means were determined by Duncan’s multiple range tests at a level of *p* < 0.05.

## 3. Results and Discussion

### 3.1. Yield and Levels of Astaxanthin

The ethanol extracted astaxanthin yield in the form of red-orange paste was 28.9 ± 3.2 mg/g shrimp shells. This result is similar to a previous study by Taksima et al. [4] who reported that extraction of astaxanthin from shrimp shells with ethanol obtained a yield of 24.7 ± 2.9 mg/g shrimp shells. The chromatographic condition used in this study gave a good resolution for the analysis of astaxanthin. The retention time of astaxanthin was 5.3 min which was found to be close to those of the astaxanthin standard. It was found that astaxanthin was the major component of shrimp extract representing about 45% among the extract (15.6 ± 0.6 mg astaxanthin/g extract). Some researchers have revealed that astaxanthin was the major carotenoid pigment with a relative percentage about 65–98% of the total carotenoid content, whereas free astaxanthin accounted for a large percentage [14,23]. For organic solvent and alcoholic extraction of astaxanthin from shrimp shells, some authors [3,5] have reported values of extract 47.86 µg/g extract and 9.27 mg/g, respectively. Thus, a higher content of astaxanthin was observed for the shrimp shells used in the present study. 

### 3.2. Antioxidant Properties of Astaxanthin Extract

#### 3.2.1. DPPH Radical Scavenging Activity

The DPPH radical scavenging assay has been commonly used to evaluate the free radical scavenging activities of antioxidants. Due to the hydrogen donating ability of antioxidants, they may reduce the free radical DPPH• to a stable form, with a decrease in absorbance at 517 nm [24]. Based on this principle, various concentrations of astaxanthin were measured for their DPPH radical scavenging activities, and the results are presented in Table 1. All samples at tested concentrations showed obvious scavenging activities on DPPH radicals in a dose-dependent manner. The lower EC_50_ value means the more powerful antioxidant capacity. Ethanol extract of astaxanthin presented antioxidant potential with the EC_50_ value of 17.5 ± 3.6 μg/mL at which the astaxanthin was comparable to BHT (17.2 ± 0.1 μg/mL). The DPPH radical scavenging activity of astaxanthin was also higher than that of the positive control, ascorbic acid (EC_50_: 19.7 ± 0.2 μg/mL). This result indicated that astaxanthin from shrimp shells had a potent antioxidant property due to its unique molecular structure, which consists of hydrocarbon with conjugated double bonds called poliene, whereas the presence of the hydroxyl (OH) and keto (C=O) endings on each ionone ring resulting in astaxanthin has hydrogen donating capability [25]. The result was consistent with Sowmya and Sachindra [23] who reported that astaxanthin from shrimp waste (*Penaeus indicus*) showed strong antioxidant activity as indicated by radical scavenging, comparable to that of the known antioxidant α-tocopherol. 

#### 3.2.2. ABTS Radical Scavenging Activity

ABTS radical scavenging assay is based on the ability of antioxidants to reduce the preformed radical ABTS with its consequent decolorization of a blue-green color in the absorbance at 734 nm [26]. In the ABTS assay, scavenging trends showed similar patterns of DPPH assay results. The astaxanthin from shrimp shells showed a concentration-dependent ABTS scavenging activity with EC_50_ value of 7.7 ± 0.6 μg/mL (Table 1). Apparently, the astaxanthin had significantly ABTS scavenging activity and more potent than ascorbic acid and BHT (EC_50_: 20.8 ± 1.1 and 15.1 ± 0.7 μg/mL, respectively). This result confirmed that astaxanthin had noticeable effect on scavenging ABTS radicals. 

#### 3.2.3. β-Carotene Bleaching (BCB) Activity

The BCB method used to evaluate the ability of a compound to prevent β-carotene oxidation by protecting it against free radicals generated during linoleic acid peroxidation. A model system undergoes discoloration in the absence of an antioxidant and it may be oxidized and subsequently, the system loses its orange color [27]. Table 1 shows the antioxidant activity of ethanol extract of astaxanthin from shrimp shells as measured by the bleaching of β-carotene. The result demonstrates a similar trend to DPPH and ABTS radical scavenging activities, where the BCB antioxidant activity increased with increasing concentration of the extract used. The EC_50_ value was found to be 15.1 ± 1.9 μg/mL. The results indicated that astaxanthin could hinder the extent of β-carotene bleaching by neutralizing the linoleate-free radical and other free radicals formed in the system. However, it was less active than the positive controls, ascorbic acid, and BHT (EC_50_: 12.5 ± 0.3 and 11.5 ± 0.1 μg/mL, respectively).

#### 3.2.4. Singlet Oxygen Scavenging (^1^O_2_) Activity

Singlet oxygen (^1^O_2_) is a non-radical ROS with one of the strongest activities. It is involved in many diseases and skin disorders. Hence, the singlet oxygen scavenging activity of natural compounds indicates their usefulness as antioxidants. The quenching of singlet oxygen of astaxanthin extract, rutin, and quercetin from the present study are shown in Table 1. Astaxanthin extract exhibited strong singlet oxygen quenching activity with EC_50_ value of 9.2 ± 0.5 μg/mL. Interestingly, the extract had significantly strong ability of ROS inhibition as compared with rutin and quercetin. This result is supported by Nishida et al. [28] who reported that astaxanthin is known to have potent singlet oxygen quenching activity and the efficiency of singlet oxygen quenching of the carotenoids has been shown to be related to the number of conjugated double bonds. As with the astaxanthin extract from shrimp shells used in the study, it exhibits strong singlet oxygen quenching activity, which indicates its usefulness as a natural antioxidant for skin and disease prevention.

### 3.3. Tyrosinase Inhibition

**Figure 1:**
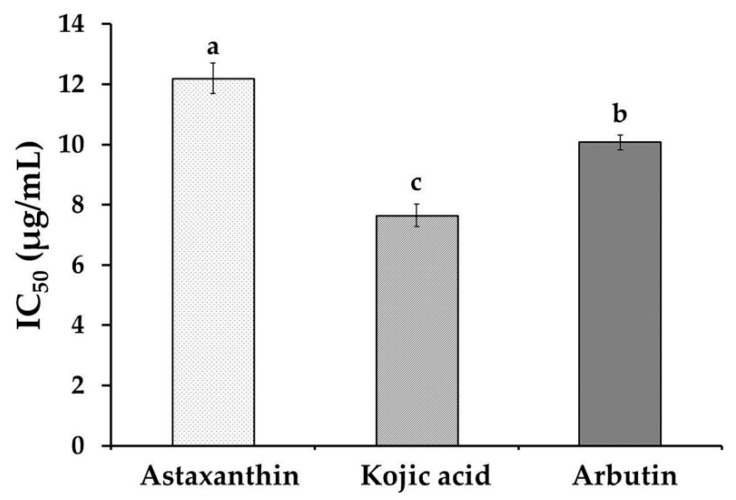
Tyrosinase inhibition activity of astaxanthin extract, kojic acid, and arbutin. Values are expressed as 50% inhibitory concentration (IC_50_). Letters with different superscripts indicate samples that are significantly different (*p* < 0.05) from each other.

Tyrosinase is an enzyme that is involved in the rate limiting step for the control of melanin production. Therefore, the inhibition of tyrosinase activity leads to induce skin whitening due to a reduction of melanin synthesis. When the tyrosinase enzyme was incubated with the astaxanthin extract, it inhibited tyrosinase activity in a dose-dependent manner at concentrations of 3–50 μg/mL. The results demonstrated that astaxanthin exhibited potent inhibitory effects against mushroom tyrosinase. The IC_50_ value of astaxanthin extracts against tyrosinase was 12.2 ± 1.5 μg/mL (Figure 1). The astaxanthin indicated relatively same inhibitory activity compared with positive control antioxidant, arbutin (IC_50_: 10.1 ± 0.2 μg/mL), however show less antityrosinase activity than kojic acid (IC_50_: 7.7 ± 0.5 μg/mL). The presence of two oxygenated groups on each ring structure, which is like a phenolic compound of astaxanthin, may be responsible for the inhibition of tyrosinase enzyme due to its ability to chelate copper in the active site [29]. This result is in concordance with the report from Xue et al. [30], that phenolic compounds such as kaempferol and quercetin were able to chelate copper atoms of tyrosinase enzyme and further inhibited tyrosinase activity. Moreover, this study showed that shrimp astaxanthin extract possessed higher tyrosinase inhibitory effect than phenolic compounds from mushroom extracts [31] and marigold flower extracts [32].

### 3.4. Cytotoxicity Assay

#### 3.4.1. Cell Viability

**Figure 2:**
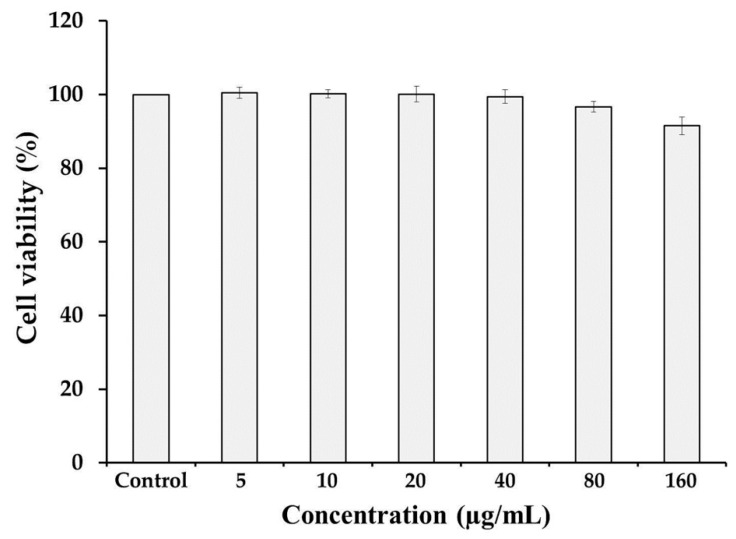
Cell viability of human dermal fibroblast cell lines exposed to astaxanthin at different concentrations for 24 h determined by 3-(4,5-dimethylthiazol-2-yl)-2,5-diphenyltetrazolium bromide (MTT) assay.

The in vitro cytotoxicity assay demonstrated obvious correlation to the toxicity in vivo [33]. MTT assay is a colorimetric method that measures the reduction of yellow tetrazolium bromide into an insoluble purple formazan product by mitochondrial succinate dehydrogenase. The amount of dye converted is directly proportional to the number of living cells [34], so it is a direct reflection of cytotoxicity. In this study, human dermal fibroblast cells (WS1) were used for toxicity evaluation. The results of cytotoxic activity of astaxanthin extract from shrimp shells are summarized in Figure 2. The viability of the cells treated with a concentration of astaxanthin in the range of 5-160 µg/mL for 24 h were not significantly different from the control, even tends to decrease with the increase in concentration. Obviously, the astaxanthin extract at all concentrations was non-toxic to human dermal fibroblast cells and gave high level of cell viability (more than 90%); thus, the half inhibition concentration (IC_50_) could not be determined. This implies that the extract does not affect the viability of the studied human skin cells. Therefore, within a reasonable dosage range, the application of astaxanthin should be safe for cosmetic development.

#### 3.4.2. Cell Morphological Characteristics

**Figure 3:**
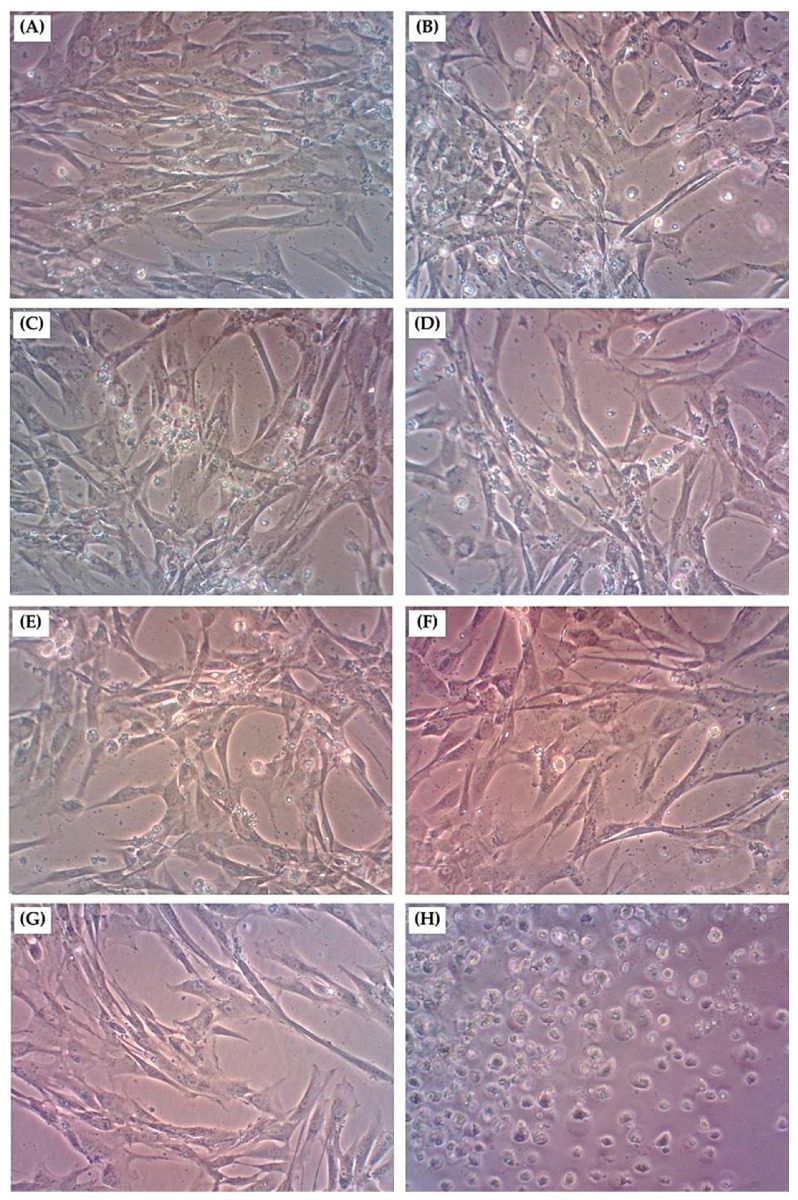
Morphological changes of human dermal fibroblast cells after exposure to astaxanthin extract for 24 h (magnification ×10). (**A**) Control cells, (**B**) cells treated with 5 µg/mL astaxanthin, (**C**) 10 µg/mL astaxanthin, (**D**) 20 µg/mL astaxanthin, (**E**) 40 µg/mL astaxanthin, (**F**) 80 µg/mL astaxanthin, (**G**) 160 µg/mL astaxanthin, and (**H**) cells treated with 100 µg/mL ascorbic acid (positive control).

To confirm astaxanthin induced cytotoxicity, we investigated the changes of cell morphology in WS1 human dermal fibroblast cells exposed to 5–160 µg/mL astaxanthin for 24 h. Images of cells under magnification are shown in Figure 3. Human dermal fibroblast cells are typically flattened or extensible shaped, adherent cells growing as a confluent monolayer (Figure 3A). For cells treated with a concentration of astaxanthin in the range of 5–160 µg/mL, no change of cell morphology could be detected compared with control cells (Figure 3B–G). Similar results were observed by other studies of fibroblast cells treated with natural extracts. Itsarasook et al. [35] reported that *Terminalia Chebula* fruit extract showed no cytoxic effect to human skin fibroblasts even at the highest concentration of 50 μg/mL because the viability of the studied cells was not affected either in the number of growth or the morphology. In this study, astaxanthin extract at all concentrations was non-toxic to human dermal fibroblasts, as shown by the cell viability higher than 90%. Meanwhile, cells induced by ascorbic acid, (the positive control) generated more pronounced cell debris and changes in morphology such as shrinkage, roundness, and detachment from the surface (Figure 3H). These results confirmed that astaxanthin from shrimp shells displayed a non-toxic effect and might be an alternative ingredient for use in cosmetics.

## 4. Conclusions

This study can be considered as the first attempt to evaluate astaxanthin from shrimp shells for possible antioxidant, antityrosinase, and cytotoxicity properties. Results from this study showed that astaxanthin possesses potent antioxidant activity and tyrosinase inhibition. Remarkably, the astaxanthin was non-cytotoxic towards human dermal fibroblast cells. We suggest that concentrations of astaxanthin in the range of 10–20 µg/mL exhibited optimal levels of antioxidant and antityrosinase activities. In addition, astaxanthin concentrations up to 160 µg/mL had no cytotoxic effects. Our findings confirmed that shrimp astaxanthin can be used as a functional ingredient in skin health products with both the efficacy and safety.

## Figures and Tables

**Table 1:** Antioxidant properties of astaxanthin extract as measured by different antioxidant assays

Samples	Antioxidant Activities (EC_50_, µg/mL)			
	DPPH	ABTS	β-Carotene Bleaching	Singlet Oxygen
Astaxanthin	17.5 ± 3.6 ^b^	7.7 ± 0.6 ^c^	15.1 ± 1.9 ^a^	9.2 ± 0.5 ^c^
Ascorbic acid	19.7 ± 0.2 ^a^	20.8 ± 1.1 ^a^	12.5 ± 0.3 ^b^	-
BHT	17.2 ± 0.1 ^b^	15.1 ± 0.7 ^b^	11.5 ± 0.1 ^b^	-
Rutin	-	-	-	55.0 ± 1.6 ^a^
Quercetin	-	-	-	50.5 ± 1.1 ^b^

^a–c^ Mean values in the same column followed by the different letters are significantly different (*p* < 0.05); BHT: Butylated hydroxytoluene.
